# When “Stay at Home” Can Be Dangerous: Data on Domestic Violence in Italy during COVID-19 Lockdown

**DOI:** 10.3390/ijerph18178948

**Published:** 2021-08-25

**Authors:** Benedetta Barchielli, Michela Baldi, Elena Paoli, Paolo Roma, Stefano Ferracuti, Christian Napoli, Anna Maria Giannini, Giulia Lausi

**Affiliations:** 1 Department of Dynamic, Clinical Psychology and Health, Sapienza University of Rome, 00185 Rome, Italy; benedetta.barchielli@uniroma1.it (B.B.); elena.paoli@uniroma1.it (E.P.); 2 Department of Psychology, Sapienza University of Rome, 00185 Rome, Italy; michela.baldi@uniroma1.it (M.B.); annamaria.giannini@uniroma1.it (A.M.G.); 3 Department of Human Neuroscience, Sapienza University of Rome, Viale dell’Università 30, 00185 Rome, Italy; paolo.roma@uniroma1.it (P.R.); stefano.ferracuti@uniroma1.it (S.F.); 4 Department of Medical Surgical Science and Translational Medicine, Sapienza University of Rome, 00189 Rome, Italy; christian.napoli@uniroma1.it

**Keywords:** quarantine, domestic abuse, coronavirus, gender-based violence, Italian context

## Abstract

The global pandemic caused by a new strain of Coronavirus has brought the Italian government to adopt quarantine, isolation, and lockdown strategies as restrictive measures to reduce the virus spread. Being forced to stay at home could significantly increase the likelihood of episodes of home-based violence and could also be accompanied by a limited possibility of complaints or defense by the victim. The present study aimed to document, through the use of newspaper articles, the characteristics of domestic violence during the lockdown period related to COVID-19 in Italy (from 9 March 2020 up to 18 May 2020) and compare the results with the same period in 2019. The results showed an increase in domestic violence during the lockdown period compared to the same period the year before and highlighted the differences between the dynamics and violent behavior between the two periods examined. The results and limitations of this research are discussed with reference to the literature.

## 1. Introduction

Domestic Violence and Abuse (DVA) is an important and widespread public health problem related to physical morbidity, psychological morbidity and mortality [1]. DVA can involve controlling, coercive, threatening, degrading and violent behavior, including sexual violence committed by a current or former spouse or intimate partner of the victim or by a person who is cohabiting with or has cohabited with the victim as a spouse or intimate partner or, also, by a family member or caretaker [2]. Therefore, the victims can be spouses, intimate/sexual partners, ex-partners, family members, children and people living together [3].

The terms DVA and Intimate Partner Violence (IPV) are used in the research field as synonymous to describe some form of abusive behavior upon one’s significant other. It is important to distinguish DVA from IPV; DVA considers different kinds of abuse, such as child and elderly abuse in a household, while IPV is a form of DVA that refers to violence by a current or former spouse or partner in an intimate relationship against the other spouse or partner [4]. These forms of violence usually involve women as the main victims, particularly for what concerns IPV [5,6]. However, the consequences of domestic violence can have important effects on the physical and mental health of female and male victims [7]. DVA and mental health are fundamentally linked, and victims experience anxiety, depression, suicidal behaviors and Post-Traumatic Stress Disorder [8]. The risk factors related to DVA are associated to the socioeconomic status; cultural background or belonging to an ethnic minority; disability and mental cognitive impairment and relationality factors such as marital status, intra-partner dependence and intergenerational transmission of violence and trauma and caregiving stress [9,10]. In addition, both geographical and social isolation may contribute to violence among people living together and, consequently, to a reduction of social support and the network, which are acknowledged as a main protective factor of DVA [11,12]. Isolation as a strategy for pandemic management can become an important risk factor for IPV, as it happened during the spread of a new strain of Coronavirus and its associated disease (COVID-19). Lockdown measures like being forced to stay at home 24 h a day with your partner or other family members significantly increases the likelihood of episodes of home-based violence and is accompanied by a limited possibility of complaints or defense by the victim [13,14,15]. Italy, from 9 March 2020 up to the 18 May 2020, enacted several highly restrictive decrees, initially limited to some areas and then extending to the whole country and lasting for three months.

Furthermore, the World Health Organization indicated some risk factors related to health emergencies and intimate partner violence (IPV), such as stress, the disruption of social and protective networks and a decreased access to protection services. Moreover, alcohol abuse is a common risk factor for domestic violence, and during COVID-19, the perpetrator of violence in isolation was more likely to abuse alcohol at home, with a likely increased risk for the whole family [16,17].

The lockdown’s economic impact is an important variable to consider for IPV. Economic difficulties can increase the stress in a households’ relationships, and this factor is combined with increased social isolation [18]. Victims have difficulty leaving an abusive relationship for an economic linkage with the partner, and the economic crisis related to the coronavirus can exacerbate the economic dependency of victim [19]. Economic dependence could also lead to forms of economic violence, a form of violence between partners that is increasingly recognized in addition to physical and sexual violence. In this case, victims of economic violence do not have the freedom to manage their financial assets, even when they may have the resources to do so, because their economic income from the abusive partner is limited [20].

Additionally relevant is the parental stress as a risk factor for domestic violence; the American Psychological Association [21] found that a large number of parents have experienced stress related to the coronavirus pandemic. Parents have managed distance/online learning, and they indicated this task as a significant source of stress. All these factors seem to have contributed to increasing the domestic violence both in Europe and worldwide since the COVID-19 outbreak began; according to Broadbury-Jones and Isham [22], in locked-down countries such as Spain and Cyprus, helpline data showed a significant increase of calls during the lockdown. Similar data has been found in the UK regarding visits to the Refuge website [23]. In Italy, however, the data differs from other countries: at the beginning of the health emergency (first half of March 2020), the calls to helplines decreased [24] and then rose exponentially. Two hypotheses can be formulated to explain this finding: firstly, forced cohabitation initially provided enough control over the victims, so that the perpetrators had no reason to exercise violence until the stressful conditions exacerbated their aggressive behavior; secondly, violent behavior occurred, but the victims had no possibility to report it because of being forced to live together with the abuser [25], defining an “emergency within an emergency”. In order to combat the phenomenon mentioned above, the Italian Ministry of Equal Opportunities developed a media campaign. From 22 March 2020, there was an exponential growth in calls to violence centers with an increase of 73% compared to the same period last year [26].

Considering these limits, we conducted research using newspaper to understand the phenomenon of domestic violence during the lockdown period; more specifically, we assumed that the isolation due to the COVID-19 lockdown in Italy would increase the number of DVA episodes. The aim of the present study was (1) to analyze the characteristics of domestic violence during the Italian lockdown related to COVID-19 (from 9 March 2020 up to 18 May 2020) and (2) compare the results with the same period in 2019 (from 9 March 2019 up to 18 May 2019).

## 2. Materials and Methods

### 2.1. Procedures

The events of violence occurring while living together were collected from the main national sources of information: the websites of two Italian press agencies and the four main Italian newspapers with national diffusion. Local press information has been used to complete the information coming from news agencies, since, from a preliminary investigation, we realized that newspapers had more exhaustive information about the violence episodes.

Events were collected during the first Italian lock down from the 9th of March 2020 and the 18th of May 2020 and during the same period of the year before. The episodes had to relate to events that could be identified as domestic violence, homicide or attempted homicide or homicide–suicide. Coding was performed using the Danger Assessment [27], a checklist identifying different forms of violence; hence, battering, aggression, threat or psychological violence, sexual violence, stalking and maltreatment were included.

Two independent researchers collected the articles in duplicate to determine whether the recovered articles met the inclusion criteria outlined above. The full texts of the articles were independently evaluated by two reviewers, who also encoded them independently; the discrepancies were resolved by an initial discussion or by a third auditor, if necessary.

All the articles concerning violence that occurred in the observation period between people living together, regardless of the relationship between them, were included. Articles of domestic violence related to episodes that occurred in previous years or in different periods from the selected one were excluded.

Since not all newspapers have adequate search engines on their websites, the research team read the national and local editions for the selected newspapers.

A dataset with the considered variables was developed. The dataset included (a, b) information about the sex, age and nationality of the perpetrator and the victim; (c) the relationship between perpetrator and victim, and the presence of children and their age and (d) the type of violence, the apparent motivation and the presence of a weapon, if any.

To conduct the research, the methodological indications that investigated homicide–suicide cases in Italy were followed [28]. Despite that, this methodology has some internal bias: first, the information contained in the newspaper depends on editorial policies and focuses more on the interest of the media; secondly, the information is partial and speculative, especially about the motivation of the offender. Even with limitations on the generalizability of the results, we compared the collected data with the official data distributed by the Ministry of Interior and the Italian National Institute of Statistics (ISTAT) to get more information on the phenomenon of domestic violence and to observe the differences between the collected data and the data available from the official reports.

### 2.2. Data Analysis

Statistical analyses were conducted using the software package SPSS (IBM Corp. Released 2017. IBM SPSS Statistics for Windows, Version 25.0. Armonk, NY, USA: IBM Corp.). An analysis of the descriptive statistics was conducted to illustrate the demographic and other selected characteristics of the domestic violence events. In addition, the comparison between the events occurred in 2019 and 2020 and was reported with a Crosstabs and *t*-test. A *p*-value < 0.05 was considered statistically significant, and a 95% confidence interval was used. Finally, two multiple logistic regression analyses were performed using the stepwise method to identify which variables might explain Violence Maltreatment before and during the COVID-19 Italian lockdown.

## 3. Results

Three hundred and forty-one episodes concerning intimate relationship violence were identified by news searches (2019: *N* = 130 and 2020: *N* = 211); a chi-square analysis reported a statistically significant difference between incidents that occurred in 2019 and 2020 (X^2^_(1, 341)_ = 19.240, *p* = 0.000); there were more cases of domestic violence during the 2020 lockdown compared to the corresponding time in 2019.

### 3.1. Perpetrators

The perpetrators were mostly males (2019: *N* = 117 and 2020: *N* = 196), and the cross-tabulation analyses did not reveal any statistically significant differences in the sex of the offenders over the two years; the ages ranged from 17 to 87 years (M = 42.68; SD = 13.66); the *t*-test analysis showed a significant difference between 2019 and 2020: in 2020, the perpetrator mean age was lower than in 2019 (t (309) = 2,334, *p* = 0.036). Finally, the nationality results showed a statistically significant difference in certain categories: there was a reduction in the number of Italian offenders (*p* < 0.05); however, no differences have been found between the categories (X^2^_(6, 341)_ = 10.995, *p* = 0.089).

No differences were found between the possible presence of psychological or psychiatric disorders or substance use in offenders between the two years (Table 1).

### 3.2. Victims

The victims were mostly females (2019: *N* = 105 and 2020: *N* = 176), and the cross-tabulation analyses did not reveal any statistically significant differences in the sexes of the victims over the two years (Table 2). The ages ranged from 1 to 97 years (M = 47.47; SD = 20.02); the *t*-test analysis showed no differences between 2019 and 2020. Finally, regarding the nationality of the victim, the chi-square analysis showed that there is a statistically significant difference between the categories of victims' nationalities, and most of them, if specified, were Italian (X^2^_(5, 341)_ = 13.555, *p* = 0.019). The results also showed a statistically significant difference in nationality; there was an increase in the number of Italian victims (*p* < 0.05).

### 3.3. Relationship and Children

A significant difference emerged between the categories (X^2^_(6, 340)_ = 22.386, *p* = 0.001) (Table 3). More specifically, in 2020, there was a significant increase in cases of violence involving both children and parents (*p* < 0.05), while the episodes between couples, siblings, roommates and exes remained stable. On the other hand, there was a statistically significant decrease in the number of episodes of violence against relatives (*p* < 0.05).

The children’s ages ranged from 3 months to 25 years old; their involvement, both as victims and witnesses of domestic violence, during the incidents was significantly higher in 2020 than 2019 (*p* < 0.05).

### 3.4. Kind of Violence, Weapon and Apparent Motivation

Regarding the type of violence perpetrated, nine categories were identified: battering, aggression with weapon, psychological violence, homicide, homicide–suicide, sexual violence, stalking, threat or maltreatment and attempted murder (Table 4).

The frequency of events between the individual categories is statistically different (X^2^_(8, 340)_ = 63.307, *p* = 0.000); in particular, between the 2019 and 2020 episodes of battering and aggression with weapons decreased, as well as murders and sexual violence, but the number of threats and maltreatment increased (*p* < 0.05). Homicide and Homicide–suicide did not show significant differences between years. When a weapon was involved, a significant increase occurred between 2019 and 2020 in stabbing (*p* < 0.05); also, a significant difference emerged between the kind of weapon categories (X^2^_(6, 119)_ = 30.121, *p* = 0.000).

Concerning the apparent motivation, the financial problems and discussions increased (*p* < 0.05) between the two considered time periods, additionally, significant differences emerged between the categories (X^2^_(7, 341)_ = 143.636, *p* = 0.000).

### 3.5. Regression Analysis

Two multiple logistic regression analyses were performed using the stepwise method. Our dependent variable was maltreatment as a form of violence; hence, we set up a new dichotomous variable (i.e., violence maltreatment) considering “maltreatment” as “yes” and all other forms of violence as “no”. The inserted variables were the age of the perpetrator, perpetrator occupation, relationship, presence of children, weapon and mainland victim.

The Wald test was used to evaluate the contribution of each individual predictor to the model. A predictor was entered into the regression equation when the probability (*p*) was 0.05. Overall, the prediction success was 84%. The prediction model did not show a fit to our observed data (χ^2^ = 4,635, *p* = 0.796). Nagelkerke’s R of 0.087 indicated a moderately strong relationship between the prediction and grouping (Table 5).

Prediction success was 62.2%. The prediction model showed no fit to our observed data (χ^2^ = 8.252, *p* = 0.409). Nagelkerke’s R of 0.118 indicated a nonsignificant relationship between the prediction and grouping (Table 6).

## 4. Discussion

Our data indicate a domestic violence increase during the first Italian lockdown period when compared to the same period in the previous year. Two hundred and eleven cases of domestic violence have been found between 9 March and 18 May 2020, while 130 cases were collected from the same period in 2019.

Consistent with the literature on the topic [29,30], victims of physical and sexual violence during the first Italian lockdown were mostly women, while the offenders were mainly men. The native countries of the offenders and the victims aggregated in macro-categories, were examined to verify their place of origin; in fact, since the perception of the origin of the perpetrators and victims is highly influenced by the media channels [31], and individuals may be affected from the media in perceiving these phenomena [32], it was decided to include this data within the research. Future studies may, once the official data collected during the lockdown period is available, compare media reports with official data and investigate the general population perception on this issue. For instance, there were no significant differences in homicides. These results are like the Italian Ministry of Interior report on voluntary homicides. In the family/affective sphere, the number of crimes decreases in 2020, with 144 homicides compared to 151 in the previous year (−5%). The number of women killed in the family/affective sphere rose from 94% in 2019 to 99% in 2020, an increase of 5%. About the period covered by this research, the report showed 41 homicides in the family environment in 2019 and 35 in 2020 with respect to 28 in 2019 and 20 in 2020 detected in the study (total of homicide and homicide + suicide). Considering that the study only covered parts of March and May, we found a similar trend to the official crime data from government institutions [33].

The results did not disclose any differences among the perpetrators, a possible explanation for this finding may be the cultural dimension of gender-based violence: domestic violence is not based on specific events but is the result of cultural and social values that cross all age groups and is the result of forms of male control over the other genders. Aggressive behaviors can be triggered by multiple and different factors and not only by substance abuse or psychological reasons. In fact, substance abuse is considered an important risk factor as it seems to increase the frequency of abuse violence and to murder between partners [34], but a causal relationship between domestic violence and substance use; abuse and dependence has not been identified in the literature [35]. Additionally, mental health was linked with an increased frequency of psychological and physical aggressions [36].

The only data that seems to have changed regarding the victim–offender relationship are those between relatives and between parents and children, which, respectively, decreased and increased. It may suggest that this finding is due to the different family organization. The time spent with children has increased significantly, considering the hours generally spent at school or at work that are now spent within the home, and this could explain the increase in episodes involving parents and children.

In relation to the above condition, increased economic insecurity could have increased the stress levels of caregivers and the probability of the use of violence against children and others within the home and reduced access to school resources contributed to increase the contact of children with violent caregivers [37]. Another risk factor is the perpetrators' previous history of violence, but these are often not reported in newspaper articles and were not found in this research.

On the other hand, the reduction of aggression against the relatives could be explained by the increased support obtained during the lockdown from the extended family. Research on this topic showed how the extended family might reduce the effects of psychosocial stress [38,39]. According to Marques and colleagues [40], during the COVID-19 crisis, the relational level may have changed due to multitasking also also increase the contact time and tensions, both in adults and young people, mainly due to restrictions on mobility and opportunities to meet people other than one’s own family.

The increase in financial motives and discussions as the reason for the violent episodes are in line with the expectations; the stressful conditions in which people have lived due to the lockdown may have given rise to more discussion; moreover, during the lockdown, many working conditions have changed: several people have stopped their work for a few months, receiving welfare that are usually lower than their usual salary. Some people might tend to act impulsively when facing conditions perceived as stressful, and aggressive behavior can be unplanned and exaggerated [41]; moreover, when experiencing an unpleasant emotion under stress, the cognitive emotion regulation could fail [42].

An interesting aspect that emerged from the data related to the forms of violence against the victims. Despite the increase in violence, in fact, most of the cases were threats and maltreatment, rather than physical and sexual violence, murder and weapon assaults. Aggression is generally known as a violent feeling aiming to inflict harm [43] but can also occur as thoughts, emotions and behaviors, or a tendency toward hostility [43]. According to the Istat data, reports of physical assault to help the baseline during lockdown increased by 9.3% compared with the same period in 2019; reports of the psychological abuse also increased 5.3% [26]; these data clearly showed how the violence incidents have increased but also highlight the gaps between the data reported by newspapers and the data collected from the calls to the Women’s Violence Hotline.

## 5. Conclusions

The information available from newspapers is sometimes limited for the editorial policies of the newspapers themselves. This limit also emerges from the logistic regressions carried out to compare the two periods examined: to assess which factors affected domestic violence, those that are recognized as risk factors for domestic violence (e.g., romantic relationship, presence/absence of children and employment status) were considered as predictors. The results from the 2019 data showed the presence of the children as the only predictor of domestic violence; while the type of relationship and the weapon seemed to be predictive factors on the data collected during the Italian lockdown. The narrative and detail selection in the newspapers is certainly a factor to be taken into account, but it can also be hypothesized that the type of relationship (IPV or DV) seems to play a more important role during the lockdown phase: a lack of social support, forced cohabitation, are all factors that may increase the perceived stress [44] and, consequently, may have led to the exacerbation of violent situations [15].

This present study also used a cross-sectional and observational design; therefore, no assumptions of causation can be made.

Despite the limitations of this study, the observed data has important potential implications at a social and psychological Level: the data within the news and the way it is reported can have an impact on the perception of the phenomenon by the news recipient. These findings may be useful in training newsrooms to provide data that is in line with the actual prevalence of the phenomenon and that communicates its importance and sensitivity.

## Figures and Tables

**Table 1:** Information about the perpetrators by years.

Perpetrator	Year								
	2019				2020			X^2^	*p*
		Count	Residual	Standardized Residual	Count	Residual	Standardized Residual		
Gender	Female	13 ^a^	2.7	0.8	14 ^a^	−2.7	−0.7	1.220	<0.05
	Male	117 ^a^	−2.7	−0.2	196 ^b^	2.7	0.2		
	Total	130			220				
Nationality	Italy	68 ^a^	9.7	1.3	85 ^b^	−9.7	−1.0	10.995	<0.05
	Europe	19 ^a^	2.6	0.6	24 ^a^	−2.6	−0.5		
	Asia	4 ^a^	−1.0	−0.4	9 ^a^	1.0	0.3		
	Africa	7 ^a^	−1.0	−0.4	14 ^a^	1.0	0.3		
	South America	4 ^a^	1.3	0.8	3 ^a^	−1.3	−0.6		
	Unknown	28 ^a^	−10.9	−1.7	74 ^b^	28.1	4.1		
	Foreign (not specified)	0 ^a^	−0.8	−0.9	2 ^a^	0.8	0.7		
	Total	130			211				
Psychiatric Diagnosis	Psychosis	0 ^a^	−1.1	−1.1	3 ^a^	1.1	0.8	5.640	<0.05
	Affective Disorder	4 ^a^	1.3	0.8	3 ^a^	−1.3	−0.6		
	Other	4 ^a^	1.7	1.1	2 ^a^	−1.7	−0.9		
	Not Specified	122 ^a^	−0.15	−0.1	202 ^a^	1.5	0.1		
	Request Assessment	0 ^a^	−0.4	−0.6	1 ^a^	0.4	0.5		
	Total	130			211				
Substance abuse	Yes	29 ^a^	−5.3	−0.9	61 ^a^	5.3	0.7	1.886	<0.05
	No	1 ^a^	0.2	0.3	1 ^a^	−0.2	−0.2		
	Not specified	100 ^a^	5.1	0.5	149 ^a^	−5.1	−0.4		
	Total	130			211				

Each superscript letter ^a,b^ denotes a subset of the YEAR categories whose column proportions do not differ significantly from each other at the 0.05 level.

**Table 2:** Information about the victims by years.

Victim	Year								
	2019				2020			X^2^	*p*
		Count	Residual	Standardized Residual	Count	Residual	Standardized Residual		
Gender	Female	105 ^a^	−2.1	−0.2	176 ^a^	2.1	0.2	0.388	<0.05
	Male	25 ^a^	2.1	0.4	35 ^a^	−2.1	−0.3		
	Total	130			211				
Nationality	Italy	62 ^a^	10.9	1.5	72 ^b^	−10.9	−1.2	13.555	<0.05
	Europe	14 ^a^	4.5	1.4	11 ^b^	−4.5	−1.1		
	Asia	4 ^a^	0.2	0.1	6 ^a^	−0.2	−0.1		
	Africa	2 ^a^	−0.7	−0.4	5 ^a^	1.3	0.7		
	South America	1 ^a^	−1.3	−0.9	5 ^a^	1.3	0.7		
	Unknown	47 ^a^	−13.6	−1.7	112 ^b^	13.6	1.4		
	Total	130			211				

Each superscript letter ^a,b^ denotes a subset of the YEAR categories whose column proportions do not differ significantly from each other at the 0.05 level.

**Table 3:** Relationship and children involvement in the episodes.

Relationship and Children	Year								
	2019				2020			X^2^	*p*
		Count	Residual	Standardized Residual	Count	Residual	Standardized Residual		
Relationship	Parents	24 ^a^	−8.1	−1.4	60 ^b^	8.1	1.1	22.386	<0.001
	Couple	85 ^a^	1.3	0.1	134 ^a^	−1.3	−0.1		
	Roomates	4 ^a^	−0.6	−0.3	8 ^a^	0.6	0.2		
	Sibilings	2 ^a^	−1.1	−0.6	6 ^a^	1.1	0.5		
	Relatives	8 ^a^	4.6	2.5	1 ^b^	−4.6	−1.9		
	Exes	4 ^a^	2.1	1.5	1 ^a^	−2.1	−1.2		
	Nonspecified	3 ^a^	1,9	1,7	1 ^b^	−1,9	−1,4		
	Total	130			211				
Children Involvement	Witnessed the violence	17 ^a^	−9.2	−1.8	36 ^b^	9.2	1.8	17.511	<0.001
	Assaulted	21 ^a^	3.7	0.9	14 ^a^	−3.7	−0.9		
	Unknown	12 ^a^	5.6	2.2	1 ^b^	−5.6	−2.2		
	Total	50			51				

Each superscript letter ^a,b^ denotes a subset of the YEAR categories whose column proportions do not differ significantly from each other at the 0.05 level.

**Table 4:** The characteristics of the episodes.

Violence Characteristics	Year								
	2019				2020			X^2^	*p*
		Count	Residual	Standardized Residual	Count	Residual	Standardized Residual		
Kind of violence	Battering	38 ^a^	10.1	1.9	35 ^b^	−10.1	−1.5	63.307	<0.001
	Aggression	17 ^a^	8.2	2.8	6 ^b^	−8.2	−2.2		
	Threat/psychological violence	3 ^a^	−2.0	−0.9	10 ^a^	2.0	0.7		
	Homicide	18 ^a^	5.4	1.5	15 ^b^	−5.4	−1.2		
	Homicide–suicide	10 ^a^	4.3	1.8	5 ^b^	−4.3	−1.4		
	Sexual violence	9 ^a^	4.4	2.1	3 ^b^	−4.4	−1.6		
	Stalking	3 ^a^	1.5	1.2	1 ^a^	−1.5	−09		
	Maltreatment	28 ^a^	−28.2	−3.8	119 ^b^	28.2	3.0		
	Attempted murder	4 ^a^	−3.6	−1.3	16 ^a^	3.6	1.0		
	Total	130			210				
Weapon	Yes	60 ^a^	14.7	2.2	59 ^b^	−14.7	−1.7	11.897	<0.001
	No	69 ^a^	−14.7	−1.6	151 ^b^	14.7	1.3		
	Total	129			210				
If weapon	Firearm	6 ^a^	−0.6	−0.2	7 ^a^	0.6	0.2	1.886	<0.05
	Cutting/stabbing	31 ^a^	−9.3	−1.5	49 ^b^	9.3	1.5		
	Strangulation	7 ^a^	3.5	1.8	0 ^b^	−3.5	−1.9		
	Mixed means	14 ^a^	6.9	2.6	0 ^b^	−6.9	−2.6		
	Fire	0 ^a^	−0.5	−0.7	1 ^a^	0.5	0.7		
	Drugs	0 ^a^	1.0	1.0	0 ^a^	−1.0	−1.0		
	Other	0 ^a^	−1.0	1.0	2 ^a^	1.0	1.0		
	Total	130			211				
Apparent motivation	Romantic Jealousy	10 ^a^	3.9	1.6	6 ^b^	−3.9	−1.2	143.636	<0.001
	Family Stressors	15 ^a^	6.6	2.3	7 ^b^	−6.6	−1.8		
	Discussion	35 ^a^	−26.4	−3.4	126 ^b^	26.4	2.6		
	End of Relationship	12 ^a^	4.0	1.4	9 ^a^	−4.0	−1.1		
	Financial Problems	0 ^a^	−10.7	−3.3	28 ^b^	10.7	2.6		
	Not Specified	0 ^a^	−9.1	−3.0	24 ^b^	9.1	2.4		
	Other	11 ^a^	2.6	0.9	11 ^a^	−2.6	−0.7		
	Total	130			211				

Each superscript letter ^a,b^ denotes a subset of the YEAR categories whose column proportions do not differ significantly from each other at the 0.05 level.

**Table 5:** Logistic regression model predicting the violence maltreatment before the COVID-19 lockdown.

	Unstandardized Coefficients (B)	S.E.	Wald	df	Sign.	Exp(B)
Age Perpetrator	−0.015	0.018	0.671	1	0.413	0.985
Perp. Occupation	1.311	1.094	1.436	1	0.231	3.710
Relationship	−0.236	0.425	0.307	1	0.579	0.790
Children’s Presence	−1.027	0.432	5.661	1	0.017 *	0.358
Weapon	0.284	0.416	0.468	1	0.494	1.329
Mainland Victim	−0.484	0.417	1.343	1	0.246	0.617
Constant	−1.363	1.490	0.837	1	0.360	0.256

Note. * Significant at the 0.05 level (two tailed test).

**Table 6:** Logistic regression model predicting the violence maltreatment during the COVID-19 lockdown.

	Unstandardized Coefficients (B)	S.E.	Wald	df	Sign.	Exp(B)
Age Perpetrator	−0.008	0.012	0.446	1	0.504	0.992
Perp. Occupation	0.568	0.574	0.979	1	0.323	1.765
Relationship	0.826	0.306	7.281	1	0.007 **	2.284
Children’s Presence	0.242	0.338	0.509	1	0.475	1.273
Weapon	1.006	0.338	8.856	1	0.003 **	2.735
Mainland Victim	0.237	0.306	0.601	1	0.438	1.268
Constant	−1.893	0.875	4.680	1	0.031 *	0.151

Note. * Significant at the 0.05 level (two tailed test); ** Significant at the 0.1 level (two tailed test).

## Data Availability

Data are available upon request.
